# CCR2 antagonism leads to marked reduction in proteinuria and glomerular injury in murine models of focal segmental glomerulosclerosis (FSGS)

**DOI:** 10.1371/journal.pone.0192405

**Published:** 2018-03-21

**Authors:** Zhenhua Miao, Linda S. Ertl, Dale Newland, Bin Zhao, Yu Wang, Xiaoping Zang, James J. Campbell, Xiaoli Liu, Ton Dang, Shichang Miao, Antoni Krasinski, Sreenivas Punna, Yibin Zeng, Jeffrey McMahon, Penglie Zhang, Israel F. Charo, Thomas J. Schall, Rajinder Singh

**Affiliations:** ChemoCentryx, Inc., Mountain View, CA, United States of America; University of Utah School of Medicine, UNITED STATES

## Abstract

Focal segmental glomerulosclerosis (FSGS) comprises a group of uncommon disorders that present with marked proteinuria, nephrotic syndrome, progressive renal failure and characteristic glomerular lesions on histopathology. The current standard of care for patients with FSGS include immunosuppressive drugs such as glucocorticoids followed by calcineurin inhibitors, if needed for intolerance or inadequate response to glucocorticoids. Renin-angiotensin-aldosterone (RAAS) blockers are also used to control proteinuria, an important signature of FSGS. Existing treatments, however, achieved only limited success. Despite best care, treatment failure is common and FSGS is causal in a significant proportion of end stage renal disease. Thus, an unmet need exists for novel disease modifying treatments for FSGS. We employed two widely-used murine models of FSGS to test the hypothesis that systemic inhibition of chemokine receptor CCR2 would have therapeutic benefit. Here we report that administration CCX872, a potent and selective small molecule antagonist of CCR2, achieved rapid and sustained attenuation of renal damage as determined by urine albumin excretion and improved histopathological outcome. Therapeutic benefit was present when CCX872 was used as a single therapy, and moreover, the combination of CCX872 and RAAS blockade was statistically more effective than RAAS blockade alone. In addition, the combination of CCR2 and RAAS blockade was equally as effective as endothelin receptor inhibition. We conclude that specific inhibition of CCR2 is effective in the Adriamycin-induced and 5/6 nephrectomy murine models of FSGS, and thus holds promise as a mechanistically distinct therapeutic addition to the treatment of human FSGS.

## Introduction

Focal segmental glomerulosclerosis (FSGS) comprises a group of uncommon disorders that present with marked proteinuria, nephrotic syndrome, progressive renal failure and glomerular lesions characterized by podocyte loss and glomerular sclerosis [1, 2]. The current standard of care for primary (idiopathic) disease includes high dose corticosteroids and/or immunosuppressants. When FSGS is secondary to other disorders, supportive management includes RAAS blockers [3–5] and optimal control of contributing factors. Despite best care, treatment failure is common, and FSGS is causal in at least 4% of all end stage renal disease. Clearly, a critical unmet medical need exists for more effective therapeutic approaches [6]. Although the pathophysiology of FSGS is not well-understood, several lines of evidence support a mechanistic role for the chemokine MCP-1 (also known as CCL2) and its receptor, chemokine receptor 2 (CCR2) in FSGS.

There is a positive correlation between increased levels of urinary MCP-1 and the degree of proteinuria associated with FSGS in both pediatric and adult patients [7, 8]. Evidence for a direct causative role comes from [9], who reported that a well-characterized polymorphism in MCP-1 (MCP-1 2518 A/G) causes increased protein expression. This increased MCP-1 expression is associated with greater risk of renal failure in both FSGS and IgA nephropathy patients. Podocytes, which are key players in pathologies involving proteinuria, directly express CCR2 and cultured human podocytes respond to MCP-1 in migration and cell-proliferation assays [10, 11].

Diabetic nephropathy, another disease characterized by progressive renal failure, also presents with significant proteinuria and loss of podocytes [12–14]. A small molecule CCR2 antagonist, CCX140-B, achieved a decreased and sustained reduction of proteinuria in diabetic nephropathy patients for 52 weeks of treatment [14].

Based on the above findings, we asked whether CCR2 inhibition could be beneficial in two well-established murine models of FSGS. In one of these models, Adriamycin induces proteinuria and segmental glomerulosclerosis after a single infusion [15]. In the other model, FSGS-like disease is induced by partial nephrectomy [15, 16]. Here we report that a CCR2 selective small molecule antagonist markedly reduced proteinuria and improved renal function in both of these widely used murine FSGS models, both as a single agent and when given in combination with RAAS and/or endothelin receptor antagonists.

## Materials and methods

### Cells and reagents

WEHI-274.1 cells were from ATCC (Rockville, MD). Human monocytes, neutrophils and lymphocytes were isolated from healthy volunteers (Stanford Blood Center, Palo Alto, CA) using MACS separation reagents (Miltenyi, Germany). The CCR2 antagonist CCX872 was discovered and synthesized at ChemoCentryx and stored as a dry powder until the time of formulation for *in vivo* use. The compound was formulated in 1% hydroxylpropyl methylcellulose (HPMC) (Sigma-Aldrich, St Louis, MO) in water for subcutaneous (s.c.) injection at the indicated concentration. Candesartan (AK Scientific, Union City, CA) and its vehicle were dosed orally once daily at 5 mg/kg in water. Recombinant chemokines were acquired from R&D Systems (Minneapolis, MN). [125I]-CCL2 was from PerkinElmer (Boston, MA). Human plasma and mouse plasma were from Bioreclamation (Hicksville, NY).

### In vitro experiments

Chemotaxis, calcium mobilization, and radio-ligand binding assays were conducted as previously described [17, 18]. Inhibition values (IC_50_) were calculated using non-linear regression with a one-site competition model (GraphPad Prism, GraphPad Software, La Jolla, CA). Urinary albumin was measured by ELISA (Bethyl Labs, Montgomery, TX). Urinary and serum creatinine was measured by LC-MS/MS at ChemoCentryx. The urinary albumin excretion rate (UAER) was calculated as micrograms per 24 h. Blood urea nitrogen (BUN) was measured by Antech Diagnostics (Morrisville, NC). Serum cystatin C was measured by ELISA (R&D Systems, Minneapolis, MN). The albumin to creatinine ratio (ACR) was calculated as micrograms of albumin per milligram of creatinine.

### Mice for pharmacokinetic and in vivo studies

Balb/c, 129s and 129X1/SvJ mice were purchased from Jackson Laboratories (Bar Harbor, Maine) and housed at the ChemoCentryx animal facility in accordance with guidelines described in the Guide and Use of Laboratory Animals of the National Research Council. All studies were approved by the ChemoCentryx Institutional Animal Care and Use Committee. All animal studies were conducted under the protocol entitled “Kidney Disease and Diabetes Models in Mice”, number CCX176-2008.

### Mouse pharmacokinetic study

CCX872 was formulated in 1% HPMC in water at 6 and 18 mg/mL concentrations, respectively. Five male 129s mice per dose group were injected s.c. with 30 and 90 mg/kg of CCX872. Blood was drawn at 0.5, 2, 5, 4, 8, 12, and 24 hours post-dosing. CCX872 plasma drug level was analyzed by LC-MS/MS at ChemoCentryx.

### Adriamycin induced nephropathy model

These experiments were performed using female Balb/c mice (Jackson Laboratories, Bar Harbor, Maine). The mice were kept on standard chow and had free access to water. At age of 10 weeks, 7.5 mg/kg Adriamycin (Selleck Chemicals, Houston, TX) or saline (control) was injected via tail vein in isoflurane-anesthetized animals (day 0). CCX872 and/or vehicle were dosed subcutaneously once daily at 90 mg/kg formulated in 1% HPMC. The RAAS blocker Candesartan (AK Scientific, Union City, CA) and its vehicle were dosed orally once daily at 5 mg/kg, formulated in water. All dosing started 2 hours prior Adriamycin challenge. The mice were housed individually in metabolic cages for quantitative collection of urinary albumin and creatinine.

### 5/6 Nephrectomy model

5/6 nephrectomy mice on the 129X1/SvJ background were obtained from Jackson Laboratories, and were received at ChemoCentryx after the surgery was performed at JAX. Under isoflurane anesthesia, two-thirds of the left kidney mass was removed at 5–6 weeks of age. Then, after 7 to 10 days, a right unilateral nephrectomy was performed. The mice were fed a standard chow and had free access to water. Three weeks after the 5/6 nephrectomy, the mice were grouped to ensure a similar starting UAER in each treatment group. CCX872 or its vehicle were dosed subcutaneously once daily formulated in 1% HPMC. The RAAS blocker Candesartan (AK Scientific, Union City, CA) and its vehicle were dosed orally once daily at 5 mg/kg formulated in water. Sparsentan, the dual-acting receptor antagonist for endothelin (A type) and angiotensin II receptors (Type 1) [19] was synthesized at ChemoCentryx and was dosed orally twice daily at 90 mg/kg formulated in 1% HPMC. The mice were housed individually in metabolic cages for quantitative collection of urinary albumin and creatinine.

### Histology and immunohistochemistry (IHC)

The kidneys were collected, fixed in formalin, embedded in paraffin, and cut into 5-μm-thick sections. Sections were stained with Haematoxylin and Eosin (H&E) (Sigma-Aldrich, St Louis, MO) or Periodic Acid Schiff (PAS) (Sigma-Aldrich, St Louis, MO) using standard protocols. Glomerular hypertrophy, mesangial expansion, glomerular sclerosis, and tubular structure were determined by examination of sections by an observer blinded to the treatment groups. Kidney podocyte staining was performed using 3-μm-thick kidney sections stained with rabbit anti-mouse monoclonal Wilms tumor protein 1 (WT-1) antibody (Abcam, Cambridge, United Kingdom) using standard methods.

### Statistical methods

Data are presented as mean with SEM for normally expressed data. Differences between treatment groups were presented with *p* values by analyzing two groups using student’s t-test using GraphPad Prism (GraphPad Software, La Jolla, CA). Each *p* value represents the difference between two groups, not a complex 4-way analysis by ANOVA test.

## Results

### CCX872 is a potent and specific antagonist of CCR2

**Fig 1:**
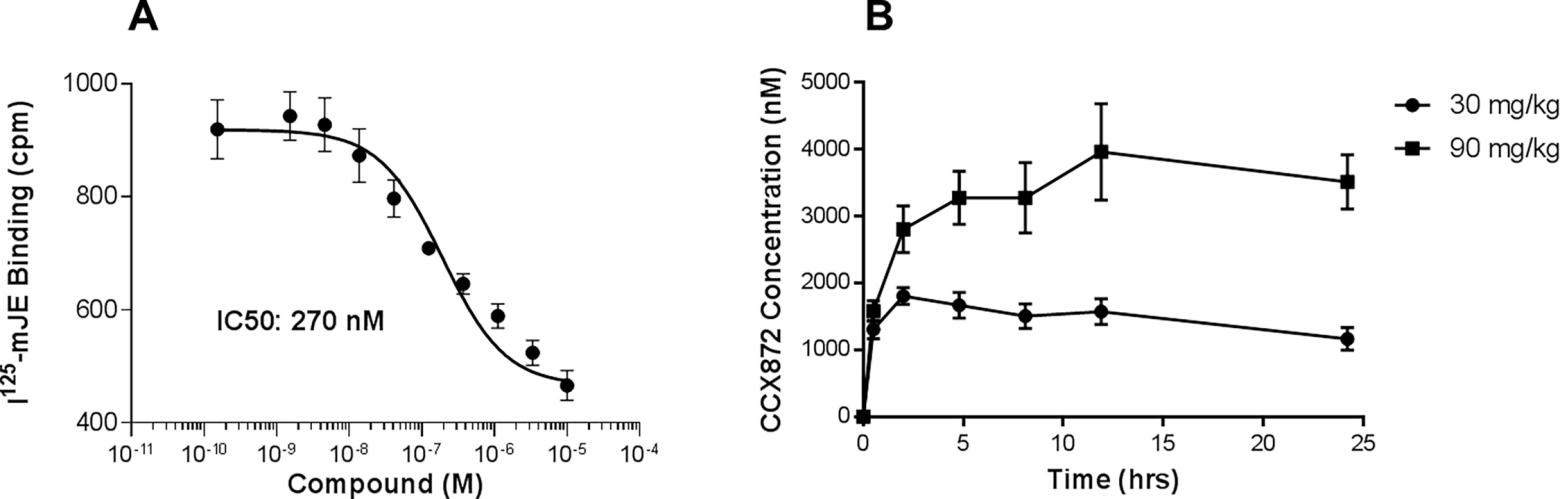
Potency and Pharmacokinetics of CCX872. A. Inhibition of Radio-labeled mJE Binding to WEHI cells. CCX872 was added at the indicated concentrations and competed with the binding of murine CCL2 (JE) to the cells, as described in Methods. The IC_50_ was determined to be 270nM (n = 8 for each data point on IC_50_ curve). Nonlinear regression (curve fit) analysis showed slope coefficient R^2^ = 0.78. B. Pharmacokinetic profile. CCX872 was administered by s.c. injection and the concentration in the blood was determined by LC-MS/MS from plasma samples.

To determine if CCR2 plays a key role in FSGS we examined the ability of CCX872 to block radio-labeled CCR2 ligand, mJE (CCL2) binding in the WEHI-274 murine monocyte cell line that endogenously expresses CCR2 [17, 18]. As shown in Fig 1A, CCX872 inhibited mJE binding in WEHI 274 cells with an IC_50_ of 270 nM (R^2^ of 0.78). To determine the selectivity of CCX872, we performed a variety of binding-competition and functional assays on cell lines that either endogenously expressed certain chemokine receptors, or were individually transfected with various receptors for other chemokines or chemotactic complement fragments. As summarized in (S1 Table), CCX872 did not inhibit any activity in a diverse selection of other receptors even at concentrations well above one micromolar. To determine if CCX872 was suitable for *in vivo* experiments, we analyzed its pharmacokinetic profile in mice. As shown in Fig 1B, a single s.c. injection of CCX872 at 90mg/kg provided a consistent level of CCX872 in the blood, with a concentration of approximately 3.5 μM, 24 hours post-dosing. We observed delayed absorption following s.c. administration of CCX872 leading to extended drug coverage. We therefore concluded that a once daily s.c. administration of CCX872 would provide more than adequate CCR2 coverage. Measurement of drug level at trough showed CCX872 concentration to be approximately ~3.5–4.1 μM at day 3 and ~5.5–6.5 μM at terminal bleed after 3–4 weeks of dosing (S2 Table), indicating slight drug accumulation during the course of multiple s.c. dosing of CCX872.

### Efficacy of CCX872 in murine models of FSGS

**Fig 2:**
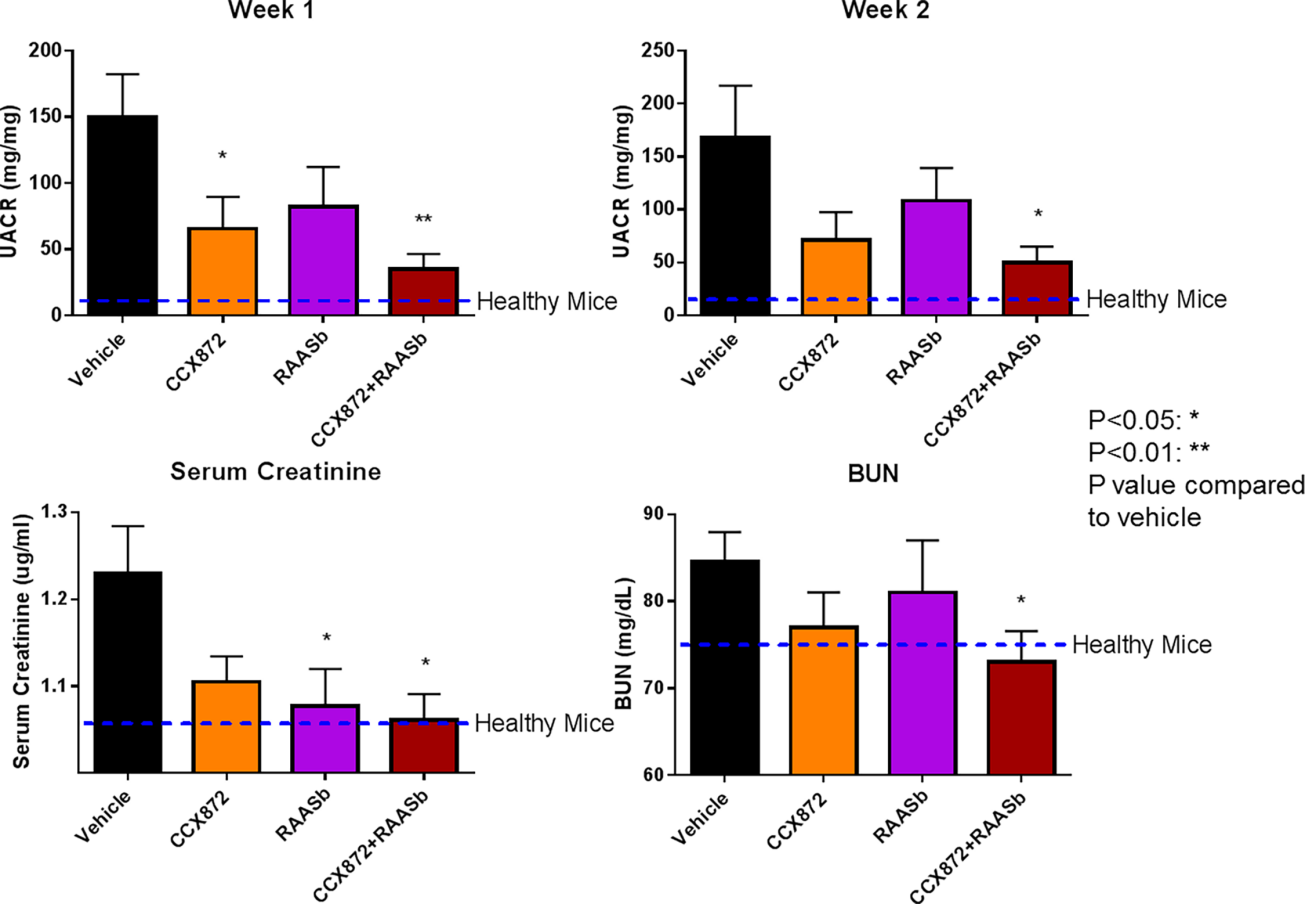
CCX872 improves renal function as assessed by UACR, serum creatinine and BUN in adriamycin challenged mice. Mice were challenged with Adriamycin as described in Methods. Test compound treatment was begun one hour prior to the Adriamycin challenge. Urine was collected for measurement of UAER (S3 Table) and creatinine at the indicated time points, and UACR was calculated as described in Methods. Serum creatinine and BUN were measured at time of terminal bleed after two weeks of treatment. Error bars represent standard error of the mean. N = 10/group at week 1, and N = 8 at week 2. *p* values were calculated on each treated group to vehicle treated animals using student’s t-test (GraphPad Prism).

To evaluate the therapeutic potential of CCX872 for FSGS we used two murine models of kidney injury. Adriamycin is an oncolytic antibiotic that induces proteinuria and glomerulosclerosis in rodents after a single infusion. Rapid and parallel reduction in UACR (mg/mg) and UAER (mg/day) by CCX872 alone, or in combination with RAAS blockade in Adriamycin nephropathy model is observed (Fig 2 and S3 Table). CCX872 as a single agent had achieved a marked reduction (65.45 ± 30.50 mg/mg compared to 150.1 ± 36.07 mg/mg with vehicle treated at week 1, 71.40 ± 26.24 mg/mg compared to 168.0 ± 48.94 mg/mg with vehicle treated at week 2) in UACR by two weeks after the Adriamycin infusion (Fig 2). Combined treatment with CCX872 and RAAS blocker achieved a statistically significant decrease in UACR with respect to vehicle (34.90 ± 12.82 compared to 150.1 ± 36.07 mg/mg with vehicle treated at week 1, 50.07 ± 14.87 compared to 168.0 ± 48.94 mg/mg with vehicle treated at week 2), accompanied by similar improvements in serum creatinine (1.06 ± 0.029 ug/ml compared to 1.229 ± 0.05 ug/ml with vehicle treated animals) and BUN levels (73.00 ± 3.59 mg/dL compared to 86.50 ± 3.73 mg/dL with vehicle treated animals) (Fig 2).

**Fig 3:**
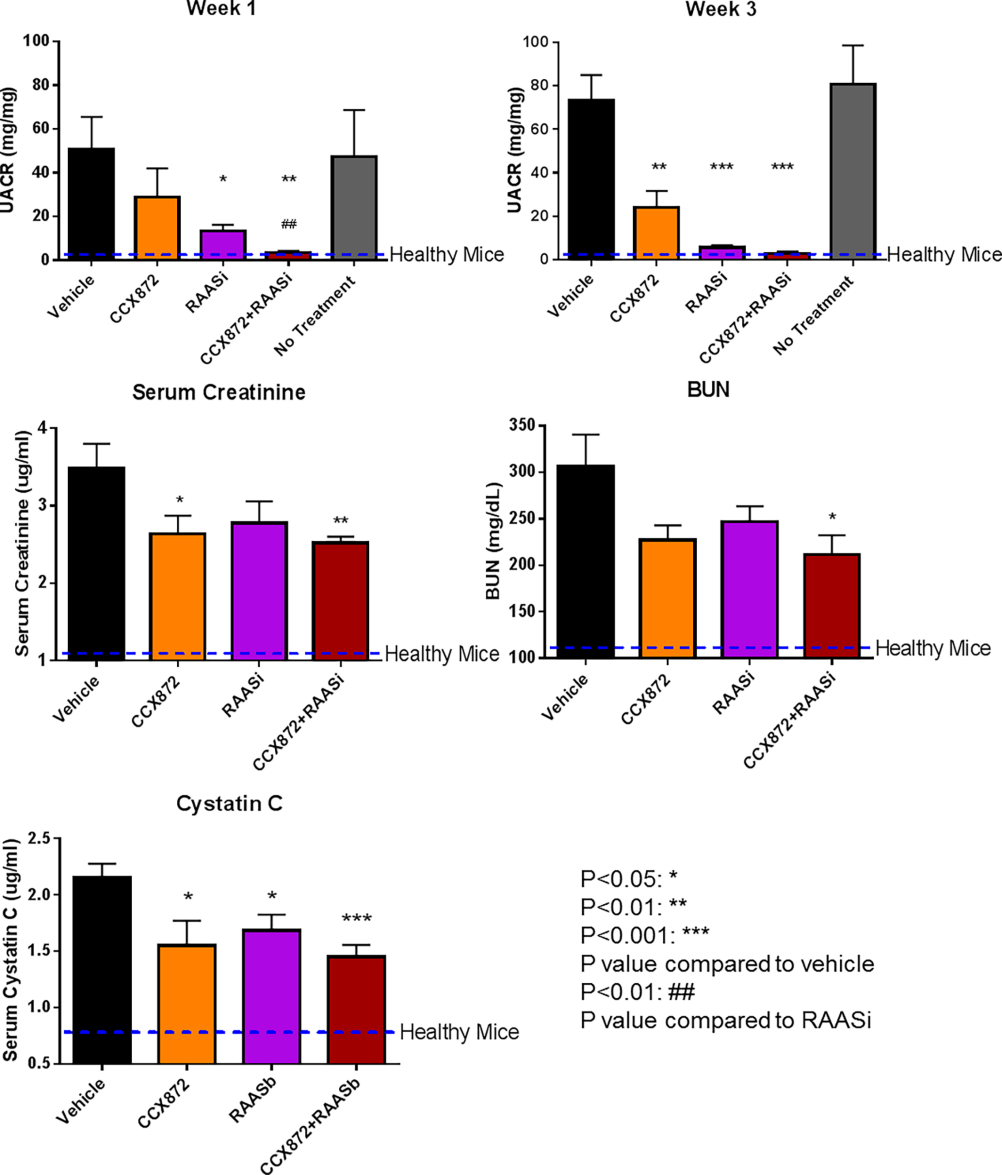
CCX872 improves renal function as assessed by UACR, serum creatinine, BUN and cystatin C in 5/6 nephrectomized mice. Mice underwent a 5/6 nephrectomy as described in Methods. Three weeks post-surgery the mice were randomized to the groups indicated above for the study period of 4 weeks. Urine was collected for measurement of albumin and creatinine at the indicated time points, and UACR was calculated as described in Methods. Serum creatinine, BUN and Cystatin C were measured at time of terminal bleed after four weeks of treatment. Error bars represent standard error of the mean. N = 10/group, and N = 6 at week 3. *p* values were calculated between each drug-treated group and vehicle treated animals (asterisk), or between CCX872/RAAS blocker combination and RAAS blocker alone (octothorpe) using student’s t-test (GraphPad Prism).

We sought to corroborate the above findings in another FSGS model mechanistically distinct from the Adriamycin approach, the 5/6 nephrectomy model. Beginning three weeks after the completion of surgery, the mice were treated with CCX872 or RAAS blocker either alone or in combination. As above, renal function was quantified by measuring UACR, serum creatinine, BUN, cystatin C levels (Fig 3) and UAER (S4 Table). Rapid and parallel reduction in UACR (mg/mg) and UAER (mg/day) by CCX872 alone, or in combination with RAAS blockade is observed (Fig 3 and S4 Table). As a mono-therapy, CCX872 markedly reduced UACR, which was apparent one week after the start of treatment (28.92 ± 13.05 mg/mg compared to 50.53 ± 15.02 mg/mg with vehicle treated at week 1) (Fig 3). This inhibition persisted throughout the study, with reductions of 72% at week three (24.00 ± 7.65 mg/mg compared to 73.36 ± 11.62 mg/mg with vehicle treated at week 3). As expected, RAAS blockade also significantly decreased the UACR (13.28 ± 2.928 mg/mg compared to 50.53 ± 15.02 mg/mg with vehicle treated at week 1, 5.679 ± 0.99 mg/mg compared to 73.36 ± 11.62 mg/mg with vehicle treated at week 3) (Fig 3). Notably, the addition of CCX872 to the RAAS blockade achieved a further, statistically significant reduction in UACR (3.48 ± 0.78 mg/mg compared to 50.53 ± 15.02 mg/mg with vehicle treated at week 1, 2.90 ± 0.95 mg/mg compared to 73.36 ± 11.62 mg/mg with vehicle treated at week 3) (Fig 3), an additive effect consistent with two distinct mechanisms of action and similar results were obtained for UACR and UAER (Fig 3 and S4 Table). CCX872 was associated with marked reductions in both creatinine (3.49 ± 0.38, 2.63 ± 0.23, 2.78 ± 0.27 and 2.51 ± 0.08 ug/ml in vehicle, CCX872, RAAS blocker or CCX872 and RAAS blocker combination treated animals, respectively) and BUN (306.7 ± 38.00, 227.0 ± 16.09, 247.0 ± 16.42 and 211.7 ± 23.03 mg/dL in vehicle, CCX872, RAAS blocker or CCX872 and RAAS blocker combination treated animals, respectively) with respect to vehicle control at the four-week time point (Fig 3). The degree to which CCX872 monotherapy reduced these parameters was greater or equal to that observed with RAAS blockade alone. Cystatin C can be used as a marker for improved renal function [20, 21]. We measured serum cystatin C level at terminal bleed. Both CCX872 and RAAS blockade significantly reduced serum cystatin C (1.55 ± 0.22 ug/mL, p = 0.030 and 1.68 ± 0.14 ug/mL, p = 0.023, respectively, compared to 2.15 ± 0.13 ug/mL for vehicle alone). The cystatin C level was further reduced in combined CCX872 and RAAS blocker treated animals (1.44 ± 0.11 ug/mL, p = 0.0006 compared with vehicle) (Fig 3). Overall the combination of CCX872 and RAAS blockade provided improved reduction of key renal markers UACR, UAER, serum creatinine and BUN (Fig 3 and S4 Table).

**Fig 4:**
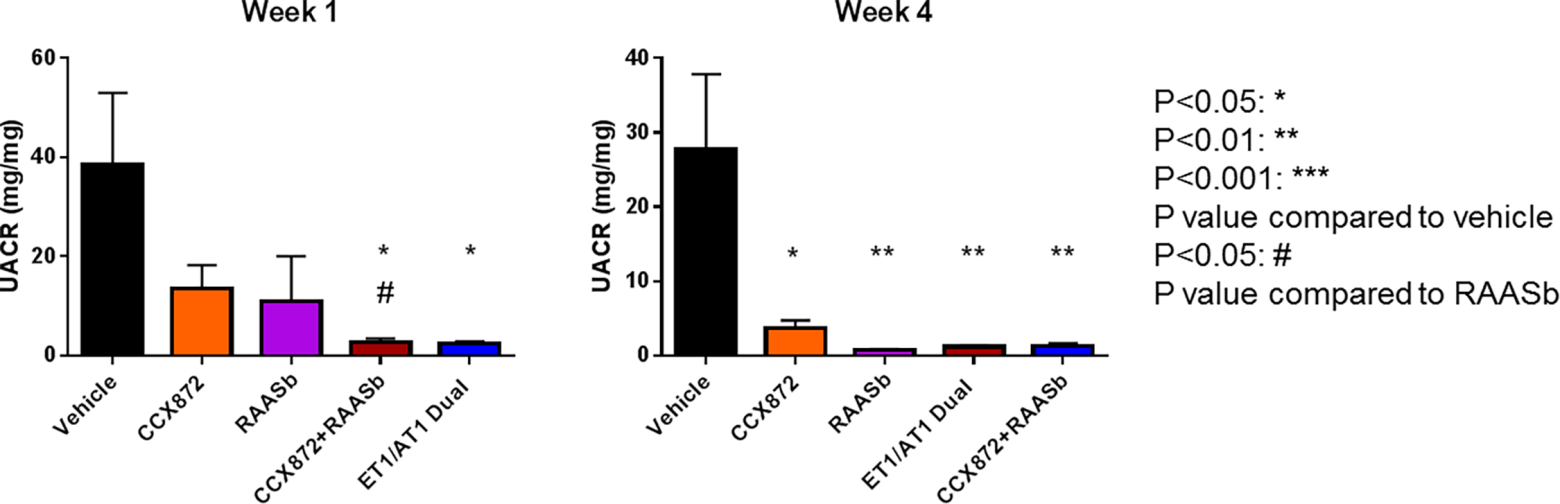
Relative efficacy of CCR2, RAAS, and endothelin receptor blockade in the 5/6 nephrectomy model as assessed by UACR (mg/mg). Mice underwent a 5/6 nephrectomy as described in Methods. Three weeks post-surgery the mice were randomized to the indicated groups for the study period of 4 weeks. Urine was collected for measurement of UACR at the indicated time points, as described in Methods. Error bars represent standard error of the mean. N = 10/group at week 1, and N = 6 at week 4. *p* values were calculated between each drug-treated group and vehicle treated animals (asterisk), or between the CCX872/RAAS blocker combination and RAAS blocker alone (octothorpe) using student’s t-test (GraphPad Prism).

Endothelin has also been implicated in FSGS, and recent clinical trials have featured a dual-acting receptor antagonist for endothelin (A type) and angiotensin II receptors (Type 1) [21]. We used the 5/6 nephrectomy model to determine how CCX872 compared to this antagonist in providing renal protection in FSGS-like disease. The combination of CCX872 and RAAS blockade was as effective as the combination of endothelin receptor inhibition plus RAAS blockade, as determined by both UACR and UAER (Fig 4 and S5 Table). Specifically, UACR was reduced by administration of CCX872 alone (64.8%) or RAAS blocker alone (73.4%), at week one (13.53 ± 4.68 mg/mg compared to 38.41 ± 14.55 mg/mg with vehicle treatment). Addition of the CCR2 antagonist to RAAS blockade further reduced the UACR (92.7% reduction vs vehicle, p = 0.02; p < 0.045 versus RAAS blockade alone), which was comparable to endothelin A/angiotensin II receptors dual antagonist (93.5% reduction vs vehicle, p = 0.019) (38.41 ± 14.55, 13.53 ± 4.68, 10.98 ± 9.02, 2.79 ± 0.65 and 2.44 ± 0.42 mg/mg in vehicle, CCX872, RAAS blocker, CCX872 and RAAS blocker combination or treated animals, respectively) (Fig 4). These results indicate that CCR2 inhibition and endothelin A/angiotensin II receptors dual antagonist inhibition is equally effective when combined with RAAS blockade in this model of FSGS.

**Fig 5:**
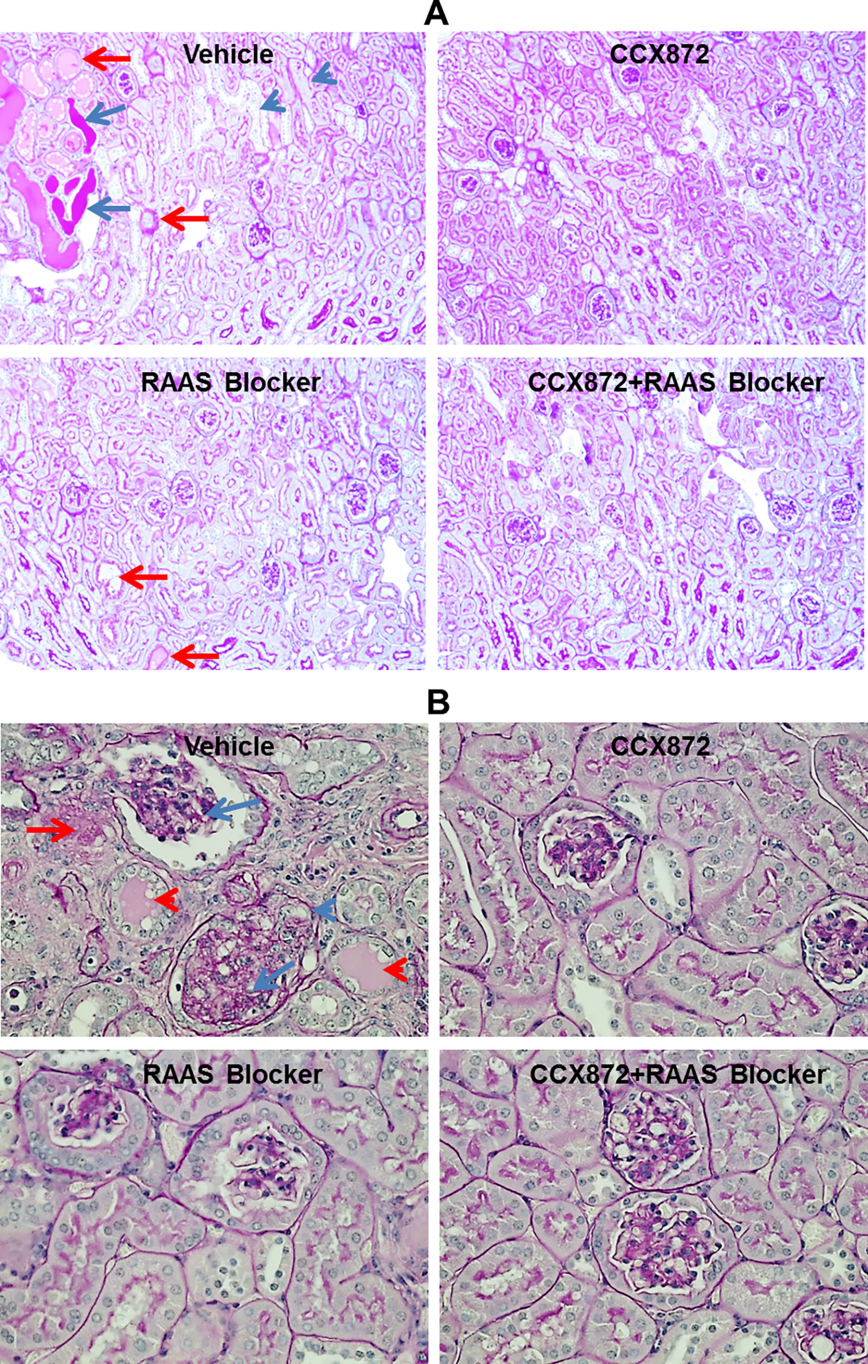
Histological analysis of kidneys from the 5/6 nephrectomy model. The kidneys (8 animals/treated groups) were harvested 5 weeks after initiation of treatment, fixed and stained as described in Methods. A. Magnification = 100X; the blue arrows indicate areas of hyaline deposition, blue arrowhead denotes tubular dilation, and red arrows denote tubular collapse. B. Magnification = 400X; the red arrows denote hyaline deposits, red arrowhead denotes tubular collapse, the blue arrows denote glomerular sclerosis, and blue arrowhead denotes mesangial expansion. Shown are representative images.

Fixed kidney sections were obtained from 5/6 nephrectomized mice for pathology. The sections were stained and evaluated to determine whether the CCX872-mediated improvements in renal function were associated with anatomical changes. As shown in Fig 5A (100x magnification) reductions in tubular dilation and hyaline deposits (with respect to vehicle control) were evident in the kidney remnants from mice treated with CCX872 alone or in combination with RAAS blockade. At higher magnification (400X), changes in the glomeruli were apparent, including decreased glomerular sclerosis, mesangial expansion, hyaline deposits and tubular collapse (Fig 5B).

**Fig 6:**
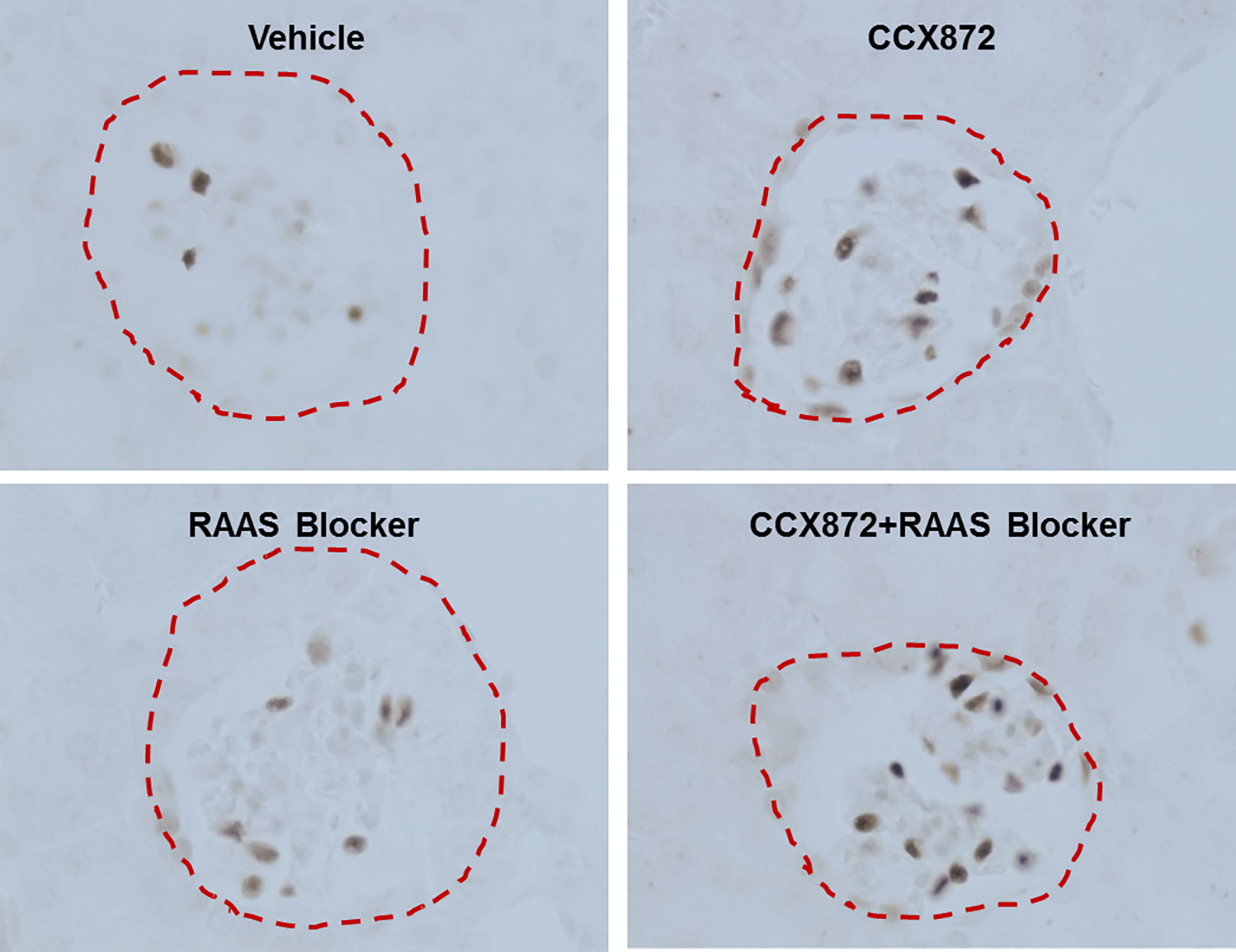
IHC analysis of kidney sections from 5/6 nephrectomy mice for changes in glomerulus size and podocyte density. The kidneys (8 animals/treated groups) were harvested 5 weeks after initiation of treatment, fixed and stained as described in Methods. All representative images of podocyte nuclear staining in various treatments were at 400X magnification. Red dashed line indicates glomeruli shape. Note the smaller glomerulus size and increased number of podocyte nuclei in the kidney section from the CCX872 and RAAS treated mice.

As increased glomerular hypertrophy and podocyte loss are characteristic of FSGS, we analyzed the effect of CCX872 treatment on glomerular size and podocyte density by staining kidney sections from 5/6 nephrectomized mice with an antibody specific for the podocyte marker Wilms tumor protein 1 (WT-1) (Fig 6). After treatment with CCX872 as single agent or in combination with RAAS blockade, glomerular size was reduced and podocyte density was increased compared with vehicle-treated mice (Fig 6).

## Discussion

FSGS is a progressive renal disease of both children and adults that rapidly progresses to end-stage renal failure when accompanied by high proteinuria. Proteinuria is the hallmark of FSGS, and proteinuria levels are positively correlated a with more rapid progression to end stage disease [6]. Agents that improve proteinuria are associated with improved prognosis [22]. Although the disease is heterogeneous and the pathophysiology complex, there is increasing evidence that antagonism of the MCP-1/CCR2 axis may be beneficial. We employed two widely-used models of FSGS, the murine 5/6 nephrectomy remnant model and Adriamycin-induced nephropathy model, to test the hypothesis that blocking CCR2 would both reduce proteinuria and improve renal function. Here we report that administration of a CCR2 antagonist was associated with rapid and sustained reduction in proteinuria and improvements in renal function as assessed by reductions in serum creatinine and BUN in both models. Significantly, CCR2 blockade improved proteinuria and renal function as a single therapeutic intervention, and had a positive additive effect when combined with RAAS blockade. Taken together, these data provide a strong rationale for investigating the effects of CCR2 inhibition in the treatment of human FSGS.

Current first-line treatment for secondary FSGS includes RAAS blockade and optimal control of the underlying disease. In primary FSGS, a trial of high dose glucocorticoids with or without immunosuppressants is recommended [23]. This intervention is effective in more than 50% of cases, but is associated with frequent relapses and significant long-term toxicity [22]. Failure to respond is associated with rapid progression to end stage renal disease requiring dialysis or renal transplant. Unfortunately, recurrence is common after transplant, consistent with speculation that circulating factors may contribute to the disease [24], leaving chronic dialysis as the only option in such cases. Thus, current therapy is both sub-optimal, and associated with serious side-effects.

One of the challenges in developing new therapeutics for FSGS has been the lack of precise animal models. A number of rodent models have been developed that recapitulate aspects of FSGS by surgically reducing total renal mass which induces podocyte damage [15]. The most frequently used model, and the one we chose for this study is the “5/6 nephrectomy” model. In this model one entire kidney, and 2/3 of the remaining kidney are sequentially removed to reduce the renal mass by 5/6 (leaving less than 20% of normal renal mass), causing hypertension and glomerular damage that histologically resembles human FSGS. To compensate for the reduced renal mass, podocytes slowly become hypertrophic, which results in reduced filtration. Sclerosis occurs as a result of abnormal filtration, and adhesions develop between the glomerular basement membrane and the Bowman’s capsule. This model thus recapitulates several key features of human FSGS, including angiotensin-driven hypertension, podocyte damage with hyperfiltration, and heterogeneous sclerosis [15]. Other models frequently employ toxins, such as puromycin or Adriamycin to reduce renal mass, but susceptibility in rodents varies by genetic background and the “pharmacological” range between disease induction and mortality tends to be narrow. Thus, although requiring significant technical expertise to execute, the 5/6 nephrectomy model has gained wide acceptance. This model is additionally validated by its responsiveness to RAAS blockers, used as add on therapy to control FSGS in the clinic.

Newer therapeutics are under development for FSGS, at least two of which have recently shown promise in human clinical trials. Sparsentan is a dual angiotensin blocker and endothelin receptor type A antagonist [19]. Sparsentan was recently evaluated in the “DUET” study, a randomized, double-blind, Phase 2 clinical trial in 109 patients with FSGS. The primary endpoint was reduction in proteinuria as compared to irbesartan. The developing company (Retrophin) recently announced that at the 8-week time point, Sparsentan achieved a statistically significant reduction in proteinuria as compared to irbesartan, thus satisfying the primary endpoint. Patients who completed the 8-week trial entered an open-label treatment program, and it will be important to determine if the decrease in proteinuria is sustained and leads to renal protection. Another therapeutic under development for FSGS, DMX-200 (Propagermanium), a combination of the organometallic compound propagermanium with irbesartan, a RAAS blocker [25]. Propagermanium is marketed for hepatitis in Japan and is reported to be an indirect CCR2 antagonist. In a clinical study combining these two drugs in 21 patients, 27% achieved a reduction in proteinuria of at least 50%. Propagermanium antagonizes receptors by targeting the enzyme phosphatidylinositol-phospholipase C, which is required for signaling by glycosyl phospholipase C (GPI) anchored proteins, some that associated with CCR2. It is very unlikely therefore that propagermanium is specific for CCR2 as there are many GPI-anchored proteins and receptors [26].

CCX872 is a selective small molecule antagonist of CCR2 and potently blocks both human and murine CCR2 with IC_50_s of 0.5 nM in human (assessed by CCL2/MCP1 induced ThP1 chemotaxis) and 270 nM in mouse (assessed by radio-labeled CCR2 binding in the murine monocyte cell line WEHI-274). CCX872 is currently in a clinical trial for safety and efficacy in pancreatic cancer. In the current study, a pharmacokinetic analysis of CCX872 in mice demonstrated a trough level of ~5.0 uM, which is at least 10-times above the *in vitro* IC_50_ for inhibition of CCR2-dependent chemotaxis. We therefore chose CCX872 to test the hypothesis that specific inhibition of CCR2 would result in decreased proteinuria and improved renal function in both the Adriamycin model and the 5/6 nephrectomy model of FSGS.

At least two lines of evidence from the current study support the notion that CCR2 is key driver of disease in FSGS. First, as noted above, specific inhibition of CCR2 resulted in a marked decrease in proteinuria. Proteinuria is one of the hallmarks of FSGS, and marked proteinuria is believed to portend declines in renal function. Indeed, recent reports for Sparsentan and DMX-200 indicate that proteinuria alone may be an acceptable Phase 3 endpoint for approval for FSGS. Second, we noted an improvement in the levels of both serum creatinine and BUN in the treated group. Improvements in these direct measures of renal function are consistent with the reduction in proteinuria, and suggest an overall beneficial effect on the glomerulus. Taken together, these data support a critical role for CCR2 in the pathophysiology of FSGS.

The role of CCR2 in FSGS has not been entirely elucidated, but such a role can be reasonably hypothesized. Chemokines are known to regulate leukocyte trafficking, and it is quite possible that a reduction in macrophage accumulation contributes to the beneficial effect. The rapidity of the response however, being clearly manifest within two weeks, suggests alternative mechanisms. In this regard, it is relevant that podocytes have been reported to express CCR2, and to undergo chemotaxis in response to MCP-1 [10, 11]. Because podocyte function is central to FSGS, it is tempting to speculate that elevated MCP-1 levels, which are widely reported to be present in FSGS [9], can directly activate podocytes via CCR2, and thus contribute to the observed rapid changes in filtration and proteinuria. Further studies will be required to test this hypothesis.

There are limitations to this study. First, although the 5/6 nephrectomy and Adriamycin models are widely used to study FSGS, they are acute models while FSGS is a chronic disease. Second, the precise mechanism whereby CCR2 inhibition leads to reduction in proteinuria and improved renal function is not known. This question is best addressed by careful studies of primary human podocyte cultures obtained from kidney biopsies of FSGS patients, and is beyond the scope of the current study. Nevertheless, following treatment of 5/6 nephrectomized mice with CCR2 inhibitor CCX872, reduction in proteinuria is associated with improvement in glomerulus size and increased podocyte density. Previously we showed CCR2 inhibition provides renal and glycemic benefits in diabetic transgenic human CCR2 knock-in mice, this improvement is associated with decreased glomerular hypertrophy and increased podocyte density [17].

In conclusion, we have utilized two murine models that recapitulate key aspects of FSGS and show that selective CCR2 inhibition results in rapid and profound decreases in proteinuria and improvements in renal function measured by creatinine and BUN. Importantly, CCR2 inhibition also improved glomerular injury as measured by improvements in tubular damage, glomerulosclerosis and podocyte density. Because CCR2 inhibition in general (and by CCX872 in particular) has proven to be safe in human clinical trials, we believe that the results presented herein provide a strong scientific rationale for evaluating the efficacy of a CCR2 antagonist in treating FSGS.

## Supporting information

S1 Table Selectivity data for CCX872 on diverse family of human chemokine and chemoattractant receptors. (DOCX)

S2 Table CCX872 concentration in Adriamycin nephropathy and 5/6 nephrectomy model. (DOCX)

S3 Table Reduction in UAER (mg/day) by CCX872 alone, or in combination with RAAS blockade in Adriamycin nephropathy model. (DOCX)

S4 Table Reduction in UAER (mg/day) by CCX872 alone, or in combination with RAAS blockade in 5/6 nephrectomy model. (DOCX)

S5 Table Reduction in UAER (mg/day) by combination of CCX872 with RAAS blockade comparing with ET1/AT2 dual inhibitor in 5/6 nephrectomy model. (DOCX)

S1 File Manuscript raw data in vitro and in vivo studies. (XLSX)
