# Supplementary material for: CCR2 antagonism leads to marked reduction in proteinuria and glomerular injury in murine models of focal segmental glomerulosclerosis (FSGS)
Source: PLoS One. 2018 Mar 21;13(3):e0192405. doi: 10.1371/journal.pone.0192405 (PMC5862408; doi:10.1371/journal.pone.0192405)
Supplement: S1 Table — (DOCX) [file pone.0192405.s001.docx]

**S1 Table.** **Selectivity Data for CCX872 on Diverse Family of Human Chemokine and Chemoattractant Receptors.**

| **Receptor** | **Assay Type** | **CCX872 Potency [nM]** |
| --- | --- | --- |
| CCR2 | ThP1 Cell Chemotaxis | 0.5 |
| CCR1 | Monocyte Chemotaxis | >10,000 |
| CCR3 | 293-CCR3 Calcium Mobilization | >10,000 |
| CCR4 | Activated Lymphocyte Calcium Mobilization | >10,000 |
| CCR5 | L1.2-CCR5 Chemotaxis | >8,000 |
| CCR6 | Activated Lymphocyte Calcium Mobilization | >8,000 |
| CCR7 | Activated Lymphocyte Calcium Mobilization | >10,000 |
| CCR8 | 293-CCR8 Calcium Mobilization | >10,000 |
| CCR9 | Molt4 Serum Chemotaxis | >10,000 |
| CCR10 | 293-CCR10 Calcium Mobilization | >10,000 |
| FPRL2 | Neutrophil Calcium Mobilization | >10,000 |
| CXCR1 | Neutrophil Calcium Mobilization | >10,000 |
| CXCR2 | Neutrophil Calcium Mobilization | >10,000 |
| CXCR3 | Activated Lymphocyte Calcium Mobilization | >10,000 |
| CXCR4 | Activated Lymphocyte Calcium Mobilization | >10,000 |
| CXCR5 | Baf3-CXCR5 Calcium Mobilization | >10,000 |
| CXCR6 | Activated Lymphocyte Buffer Chemotaxis | >10,000 |
| CXCR7 | 293-CXCR7 Radio-ligand Binding | >10,000 |
| C3aR | Neutrophil Calcium Mobilization | >10,000 |
| C5aR | Neutrophil Calcium Mobilization | >10,000 |

Shown are the IC_50_ values for inhibition of the specified responses.
