# Supplementary material for: CCR2 antagonism leads to marked reduction in proteinuria and glomerular injury in murine models of focal segmental glomerulosclerosis (FSGS)
Source: PLoS One. 2018 Mar 21;13(3):e0192405. doi: 10.1371/journal.pone.0192405 (PMC5862408; doi:10.1371/journal.pone.0192405)
Supplement: S2 Table — (DOCX) [file pone.0192405.s002.docx]

**S2 Table. CCX872 concentration in Adriamycin nephropathy and 5/6 nephrectomy model.**

| Study | Day 3 Trough Drug Level (24 hr post last dosing) | Terminal Bleed Trough Drug Level (24 hr post last dosing) |
| --- | --- | --- |
| Adriamycin Nephropathy Model | 4.10 ± 0.82 (uM) | 6.52 ± 1.21 (uM) |
| 5/6 Nephrectomy Model | 3.72 ± 0.25 (uM) | 5.65 ± 0.95 (uM) |
