# Supplementary material for: CCR2 antagonism leads to marked reduction in proteinuria and glomerular injury in murine models of focal segmental glomerulosclerosis (FSGS)
Source: PLoS One. 2018 Mar 21;13(3):e0192405. doi: 10.1371/journal.pone.0192405 (PMC5862408; doi:10.1371/journal.pone.0192405)
Supplement: S3 Table — (DOCX) [file pone.0192405.s003.docx]

**S3 Table. Reduction in UAER (mg/day) by CCX872 alone, or in combination with RAAS blockade in Adriamycin nephropathy model.**

|  | Week 1 | Week 2 |
| --- | --- | --- |
| Vehicle | 75.87 ± 21.41 | 89.99 ± 26.39 |
| CCX872 | 26.15 ± 10.39, p=0.06 | 24.20 ± 7.80, p=0.032 |
| RAAS Blocker | 39.46 ± 14.14, p=0.16 | 66.87 ± 23.99, p=0.52 |
| CCX872+RAAS Blocker | 20.39 ± 8.11, p=0.027 | 23.31 ± 8.54, p=0.030 |
