# Supplementary material for: CCR2 antagonism leads to marked reduction in proteinuria and glomerular injury in murine models of focal segmental glomerulosclerosis (FSGS)
Source: PLoS One. 2018 Mar 21;13(3):e0192405. doi: 10.1371/journal.pone.0192405 (PMC5862408; doi:10.1371/journal.pone.0192405)
Supplement: S4 Table — (DOCX) [file pone.0192405.s004.docx]

**S4 Table. Reduction in UAER (mg/day) by CCX872 alone, or in combination with RAAS blockade in 5/6 nephrectomy model.**

|  | Week 1 | Week 2 | Week 3 |
| --- | --- | --- | --- |
| Vehicle | 26.82 ± 7.82 | 17.22 ± 5.18 | 39.55 ± 5.22 |
| CCX872 | 15.68 ± 7.02, p=0.30 | 6.72 ± 1.71, p=0.07 | 11.75 ± 3.23, p=0.001 |
| RAAS Blocker | 8.27± 1.89, p=0.03 | 3.62 ± 1.63, p=0.03 | 3.32 ± 0.76, p<0.0001 |
| CCX872+RAAS Blocker | 2.04 ± 0.46, p=0.005 | 1.45 ± 0.38, p=0.009 | 1.67 ± 0.54, p<0.0001 |
| No Treatment | 31.88 ± 13.88 | 20.58 ± 12.74 | 21.08 ± 14.23 |

^1^ CCX872, 90 mg/kg

^2^ RAAS Blocker, 5 mg/kg
