# Supplementary material for: CCR2 antagonism leads to marked reduction in proteinuria and glomerular injury in murine models of focal segmental glomerulosclerosis (FSGS)
Source: PLoS One. 2018 Mar 21;13(3):e0192405. doi: 10.1371/journal.pone.0192405 (PMC5862408; doi:10.1371/journal.pone.0192405)
Supplement: S5 Table — (DOCX) [file pone.0192405.s005.docx]

**S5 Table. Reduction in UAER (mg/day) by combination of CCX872 with**

**RAAS blockade comparing with ET1/AT2 dual inhibitor in 5/6 nephrectomy model.**

|  | Week 1 | Week 2 | Week 4 |
| --- | --- | --- | --- |
| Vehicle | 20.70 ± 6.54 | 23.75 ± 5.75 | 23.26 ± 8.77 |
| CCX872 | 7.13 ± 1.72, p=0.07 | 11.03 ± 5.28, p=0.10 | 2.85 ± 3.44, p=0.02 |
| RAAS Blocker | 9.38 ± 7.70, p=0.27 | 7.72 ± 6.37, p=0.06 | 1.74 ± 2.49, p=0.02 |
| CCX872+RAAS Blocker | 2.10 ± 0.49, p=0.012 | 1.95 ± 1.43, p=0.002 | 1.54 ± 0.78, p=0.001 |
| ET1/AT2 Dual Blocker | 2.64 ± 0.75, p=0.036 | 1.54 ± 2.15, p=0.01 | 1.15 ± 1.47, p=0.001 |

^1^ CCX872, 90 mg/kg

^2^ RAAS Blocker, 5 mg/kg
